# The gut microbiota modulates differential adenoma suppression by B6/J and B6/N genetic backgrounds in *Apc*^*Min*^ mice

**DOI:** 10.1007/s00335-019-09814-3

**Published:** 2019-09-23

**Authors:** Jacob E. Moskowitz, Federica Andreatta, James Amos-Landgraf

**Affiliations:** 1 Department of Veterinary Pathobiology, University of Missouri, Columbia, MO 65201 USA; 2 Department of Comparative Biomedicine and Food Science, University of Padua, 35020 Padua, Italy; 3 Mutant Mouse Resource and Research Center, University of Missouri, 4011 Discovery Drive, Columbia, MO 65201 USA

## Abstract

Tumor multiplicity in the *Apc*^*Min*^ (Min) mouse model of CRC is a classic quantitative trait that is subject to complex genetic and environmental factors, and therefore serves as an ideal platform to study modifiers of disease. While disparate inbred genetic backgrounds have well-characterized modifying effects on tumor multiplicity, it is unclear whether more closely related backgrounds such as C57BL/6J and C57BL6/N differentially modify the phenotype. Furthermore, it is unknown whether the complex gut microbiota (GM) influences the effects of these background strains. We assessed tumor multiplicity in F1 mice generated from the original Min colony from the McArdle Laboratory at the University of Wisconsin (C57BL/6JMlcr-*Apc*^*Min*^) crossed with either C57BL/6J or C57BL/6N wild-type mice. We also used complex microbiota targeted rederivation to rederive B6NB6JMF1-*Apc*^*Min*^ embryos using surrogate dams harboring complex GMs from two different sources to determine the effects of complex GM. Both B6/J and B6/N backgrounds significantly repressed tumor multiplicity. However, the B6/N background conferred a stronger dominant suppressive effect than B6/J. Moreover, we observed that complex GM likely modulated B6/N-mediated adenoma repression such that two distinct communities conferred differential tumor multiplicity in isogenic B6NB6JMF1-*Apc*^*Min*^ mice. Although we cannot rule out possible maternal effects of embryo transfer, we show that B6/J and B6/N have modifier effects on Min, and these effects are further altered by the complex GM. Foremost, strict attention to genetic background and environmental variables influencing the GM is critical to enhance reproducibility in models of complex disease traits.

**Electronic supplementary material:**

The online version of this article (10.1007/s00335-019-09814-3) contains supplementary material, which is available to authorized users.

## Introduction

The challenges of controlling for genetic and environmental variability in the study of human diseases such as colorectal cancer (CRC) necessitate the use of animal models that enable the resolution of complex traits. The *Apc*^*Min*^ (Min) mouse model of CRC, which harbors an autosomal dominant mutation in the *Apc* tumor suppressor gene causing the development of intestinal adenomas, provides an extensively studied and quantitative platform to investigate factors contributing to disease initiation (Shoemaker et al. 1997). Due to well-known phenotypic variability across Min colonies, defined by differential adenoma multiplicity, the Min mouse is especially useful for elucidating underlying modifiers of disease susceptibility (Kwong and Dove 2009). In various studies, investigators have observed modifications of adenoma multiplicity stemming from both spontaneously occurring variants within the C57BL/6J (B6/J) strain, and through crosses with different mouse strains. Through these approaches, a number of loci that can substantially affect adenoma multiplicity were identified, and aptly termed Modifier of Min (*Mom*) loci (Baran et al. 2007; Cormier et al. 2000; Dietrich et al. 1993; Kwong and Dove 2009). Despite the contributions of these studies, the extensive genetic variance between different inbred strains results in a large amount of “background noise,” making it more difficult to resolve the numerous genetic modifiers of consequence. Thus, it may be advantageous to focus on efforts to uncover modifiers of Min on strains more closely related to B6/J. To that end, the C57BL/6N (B6/N) background is of interest as a related but divergent substrain of C57BL/6 that has now been separated from the B6/J substrain for hundreds of generations (Bryant 2011). During the course of B6/J and B6/N divergence, the two substrains have accumulated distinct genotypes and phenotypes spanning various biological systems, ranging from neurobehavioral to immune responses (Bryant 2011; Fontaine and Davis 2016). Unfortunately, these substrains are often used interchangeably, and therefore important distinctions that may strongly influence a phenotype of interest are ignored. However, a keen awareness of these distinctions could provide leverage to uncover modifiers of complex traits. To date, it is unclear whether the B6/J and B6/N substrains have distinct modulatory effects on the Min phenotype as a result of their underlying divergence.

More recently, the gut microbiota (GM) has also emerged as an important factor in complex disease traits such as inflammatory bowel disease (IBD) and CRC (Louis et al. 2014; Rubin et al. 2012). The GM was initially identified as an important factor through associative studies, where investigators linked depletion or enrichment of certain taxa to disease status (Ohigashi et al. 2013; Scanlan et al. 2008). Studies in animal models with quantitative complex disease traits have shed further light on the dynamic relationship between host and microbe. In a recent study, *il10*^−/−^ mice, which develop intestinal inflammation due to loss of immunoregulatory IL-10, were colonized with three distinct GM communities. The isogenic mice subsequently developed differing severity of disease based on GM colonization, corroborating the important role for intestinal microbial communities (Hart et al. 2017). Moreover, germ-free Min mice have a significantly lower adenoma burden than their colonized counterparts, suggesting that the GM influences the Min phenotype (Li et al. 2012). Despite indications that the GM affects the Min phenotype, further studies are required to determine how different complex communities contribute to variability across Min colonies.

In this study, we addressed two questions in an effort to further our understanding of different factors contributing to phenotypic variability across Min colonies. First, we aimed to determine how the B6/N genetic background modifies adenoma multiplicity in comparison to the B6/J strain. Furthermore, we asked whether colonization with distinct complex GM communities further modulates adenoma multiplicity. We established F1 Min cohorts using Min males from the original colony at the McArdle Laboratory of the University of Wisconsin (C57BL/6JMlcr-*Apc*^*Min*^/Mmmh abbrv. B6/JM-*Apc*^*Min*^*),* and either B6/J or B6/N wild-type females. F1 offspring from the B6/N lineage (B6NB6JMF1) were rederived using surrogates harboring two different complex GMs, such that isogenic mice were born with two distinct GMs. We describe differential adenoma suppression mediated by the B6/J and B6/N strains across GM-controlled groups, and illustrate potential maternal effects that may contribute to phenotype variability. We further demonstrate GM-mediated phenotype modulation in isogenic mice. Through these approaches, we develop a platform for identifying genetic and bacterial modifiers of the Min phenotype, and concurrently illustrate how complex variables shape complex quantitative traits in a classic cancer model.

## Results

### B6JB6JMF1-*Apc*^*Min*^ mice exhibit partial repression of the B6/JM adenoma phenotype

**Fig. 1:**
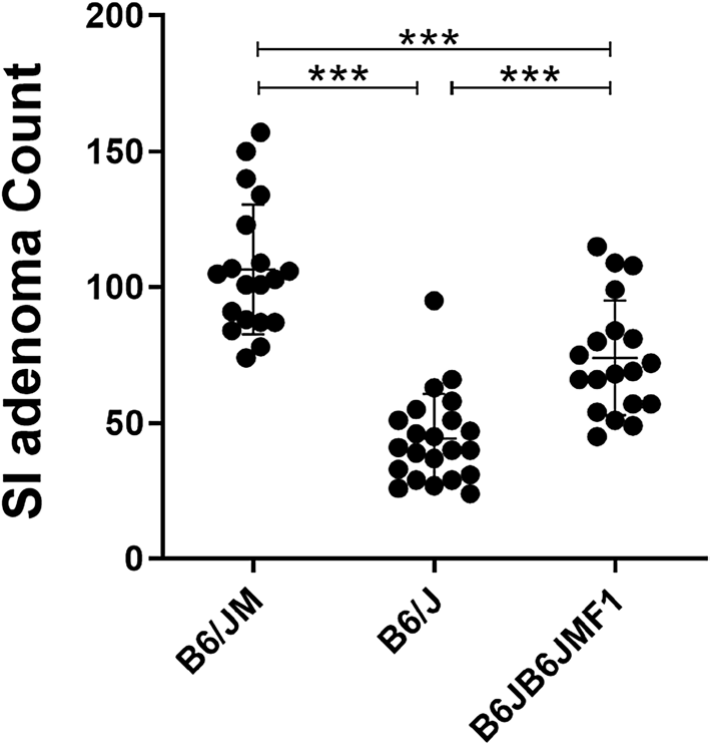
B6JB6JMF1-*Apc*^*Min*^ mice display an intermediate adenoma phenotype. Scatter plots comparing mean (± SD) small intestinal (SI) adenoma counts of the original B6-*Apc*^*Min*^ colony generated at UW McArdle Laboratory (B6/JM), B6-*Apc*^*Min*^ mice acquired from The Jackson Laboratory and maintained at University of Missouri (B6/J), and their F1 offspring (B6JB6JMF1) (B6/JM, *n* = 19; B6/J, *n* = 22; B6JB6JMF1, *n* = 19). ***p < 0.001; ANOVA with the Student Newman–Keuls method

The B6/JM-*Apc*^*Min*^ and B6/J-*Apc*^*Min*^ parental colonies were maintained as distinct colonies within the same facility at the University of Missouri. At 100 days of age, Min mice from both parental lines were humanely sacrificed for adenoma counts. We observed significantly fewer adenomas in the B6/J line (44.2 ± 16.5 adenomas) compared to the B6/JM line (106.6 ± 24.0 adenomas; *p *< 0.001) (Fig. 1). To determine the capacity for the B6/J genetic background to repress the B6/JM phenotype, wild-type female B6/J mice were mated with Min male B6/JM mice to generate B6JB6JMF1-*Apc*^*Min*^ offspring. F1 offspring were similarly sacrificed at 100 days of age for adenoma counts. We found that the F1 generation displayed an intermediate adenoma phenotype (73.9 ± 21.1 adenomas), as they developed significantly fewer adenomas than the B6/JM line and significantly more adenomas than the B6/J parents (*p *< 0.001) (Fig. 1).

### B6/N repression of adenomas is modulated by the gut microbiota

**Fig. 2:**
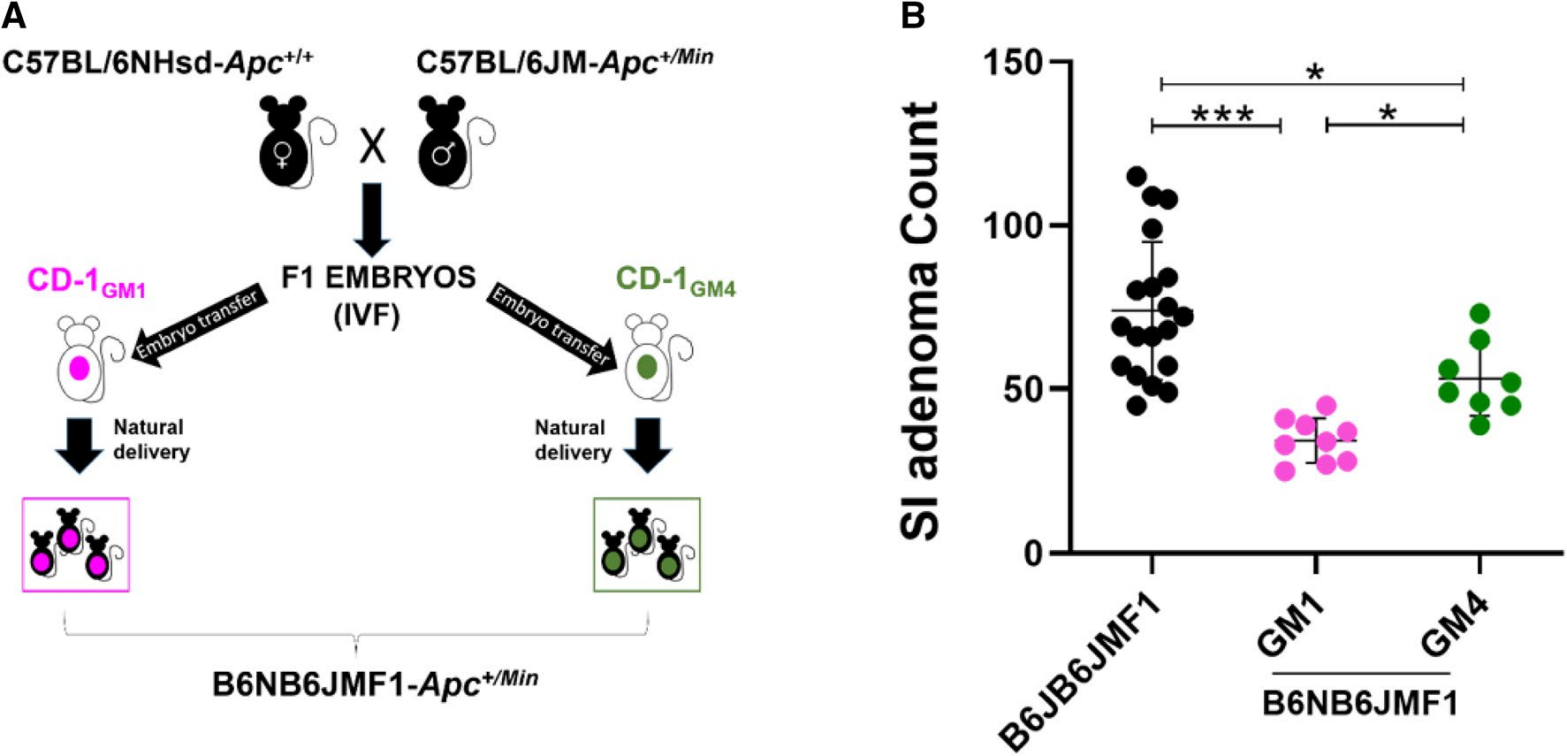
The C57BL/6NHsd genetic background and the gut microbiota modulate adenoma repression in *Apc*^*Min*^ mice. **a** F1 embryos from C57BL/6NHsd-*Apc*^+/+^ and C57BL/6JD-*Apc*^+/*Min*^ parental lines were generated via IVF, and transplanted into CD-1 surrogate dams harboring two distinct complex GM profiles; GM1 and GM4. Offspring maintain their F1 hybrid genetic lineage while inheriting a GM from respective surrogate CD-1 dams. (${\text{B6NB6JMF1-}}{Apc^{Min}}_{\text{GM1}}$, *n* = 9; ${\text{B6NB6JMF1-}}{Apc^{Min}}_{\text{GM4}}$, *n* = 8). **b** Scatter plots comparing mean (± SD) SI adenoma counts of B6JB6JMF1-*Apc*^*Min*^, ${\text{B6NB6JMF1-}}{Apc^{Min}}_{\text{GM1}}$, and ${\text{B6NB6JMF1-}}{Apc^{Min}}_{\text{GM4}}$ mice. *p < 0.05, **p < 0.01, ***p < 0.001; ANOVA with the Student Newman–Keuls method

Given the divergence between the B6/J and B6/N lineages, we first asked whether the B6/N genetic background would exert similar adenoma repression as the B6/J genetic background when crossed with the B6/JM lineage. Furthermore, we aimed to determine whether distinct complex GM communities would modulate the effects of the B6/N genetic background in the context of adenoma multiplicity. We performed complex microbiota targeted rederivation (CMTR) to establish B6NB6JMF1-*Apc*^*Min*^ mice with two distinct complex GMs; a low-richness community (GM1) and a high-richness community (GM4) using CD-1 surrogate dams with GMs that originally represented The Jackson Laboratory (GM1) or Envigo (GM4) (Fig. 2a). Of note, the B6/J and B6/JM parental colonies were independently rederived using GM1 surrogate dams and maintained as distinct colonies. At 100 days of age, both ${\text{B6NB6JMF1-}}{Apc^{Min}}_{\text{GM1}}$ and ${\text{B6NB6JMF1-}}{Apc^{Min}}_{\text{GM4}}$ groups were sacrificed for adenoma counts, and compared to B6JB6JMF1-*Apc*^*Min*^. We found that Min B6NB6JMF1 mice, regardless of GM colonization, developed significantly fewer SI adenomas than Min B6JB6JMF1 mice (Fig. 2b). We also observed a significant effect of the GM, where Min B6NB6JMF1 mice colonized with GM4 (53.1 ± 11.2 adenomas) developed more adenomas than those colonized with GM1 (34.3 ± 6.8 adenomas). Thus, the B6/N genetic background demonstrated stronger suppression of the B6/JM phenotype compared to the B6/J background. Moreover, the degree of B6/N-mediated suppression was modulated by the GM.

### Distinct GMs confer differential adenoma susceptibility

**Fig. 3:**
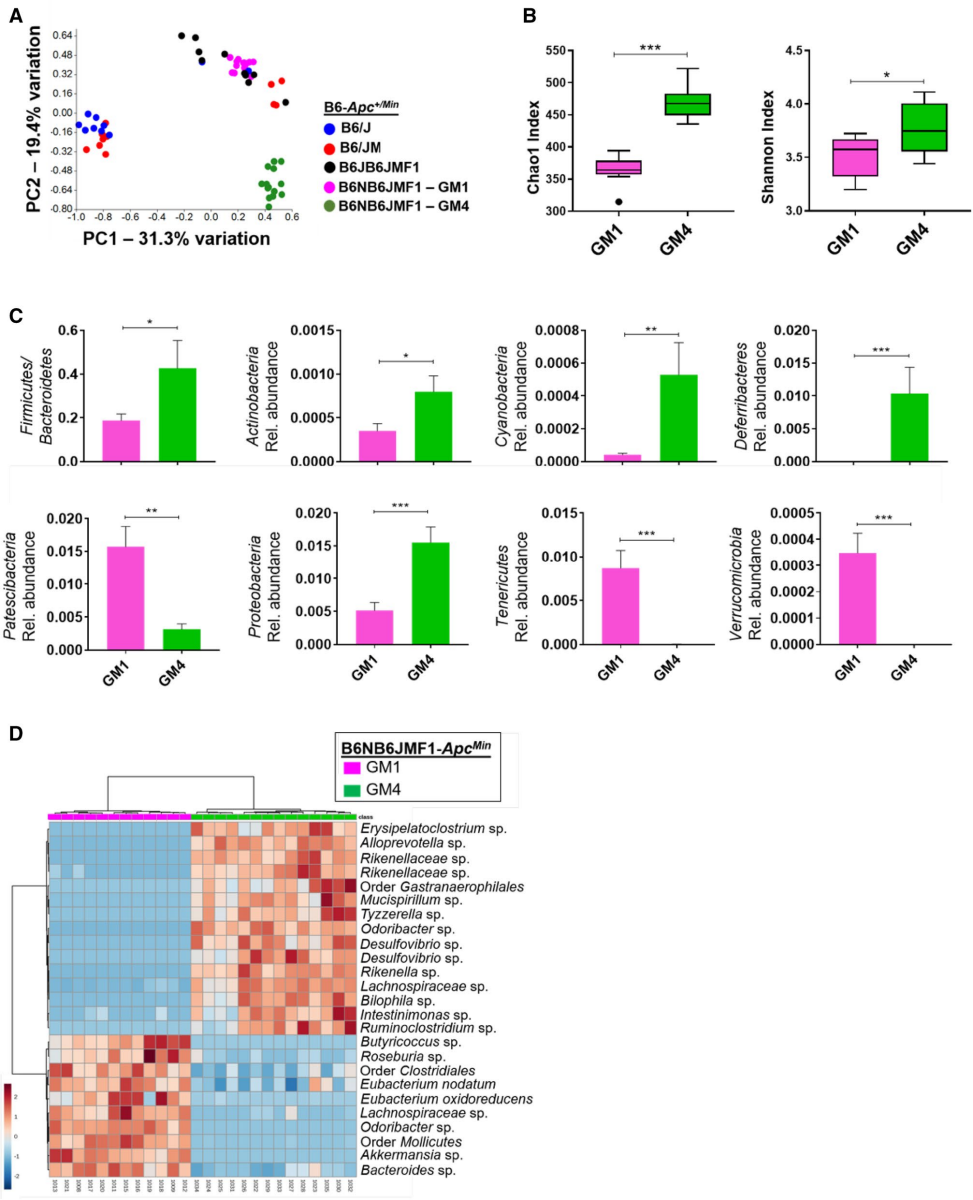
Distinct complex gut microbiota communities contribute to differential adenoma counts. **a** Principal component analysis (PCA) representing differences in β-diversity at the operational taxanomic unit (OTU) level between complex GM profiles of B6/J (*n* = 8) and B6/JM (*n* = 11) parental lines, B6JB6JMF1 offspring (*n* = 10), ${\text{B6NB6JMF1-}}{Apc^{Min}}_{\text{GM1}}$ (*n* = 13), and ${\text{B6NB6JMF1-}}{Apc^{Min}}_{\text{GM4}}$ mice (*n* = 14). **b** Differences in GM richness (Chao1 index) and α-diversity (Shannon Index) are shown with Tukey’s boxplots (GM1, *n* = 9, GM4, *n* = 8). **c** Bar charts representing relative abundances (mean ± SEM) of phyla with detected significant differences between GM1 and GM4, in fecal samples from mice at 1 month of age (GM1, *n* = 9; GM4, *n* = 8). **d** Heatmap showing 25 taxa with significantly different (FDR< 0.05) fecal relative abundances between GM1 and GM4 at 1 month. Color intensity represents fold-change of each OTU. Hierarchical clustering based on Euclidean distances (top) demonstrates clustering of samples based on GM. All statistically significant OTUs and associated fold changes are represented in Supplementary Table 1.*p < 0.05, **p < 0.01, ***p < 0.001; Student’s *t* test

Given the significant effect of the GM on adenoma numbers in B6NB6JMF1-*Apc*^*Min*^ mice, we characterized GM1 and GM4 through 16S rDNA sequencing. Fecal samples were collected from B6NB6JMF1_GM1_ and B6NB6JMF1_GM4_ mice at 1 month of age and subject to sequencing of the 16S rRNA microbial gene to determine relative abundances of all detected bacterial phyla and operational taxonomic units (OTUs). Fecal samples were also collected from a subset of the B6/J and B6/JM parental colonies and B6JB6JMF1 mice. To visualize overall GM community similarity of parental colonies and F1 mice, a Principal Component Analysis (PCA) was used (Fig. 3a). Notably, B6JB6JMF1 and B6NB6JMF1_GM1_ demonstrate similar overall GM communities, both of which are distinct from ${\text{B6NB6JMF1-}}{Apc^{Min}}_{\text{GM4}}$ mice. We noted GM variability among the B6/J and B6/JM parental colonies, such that some samples closely resembled the B6JB6JMF1 and B6NB6JMF1_GM1_ mice while others had a distinct profile. Mice colonized with GM4 had increased Chao1 and Shannon indices, indicating increased richness and diversity compared to GM1, respectively (Fig. 3b). We found that GM4 was enriched with the phyla *Actinobacteria, Cyanobacteria, Deferribacteres, Proteobacteria*, and had an increased *Firmicutes*-to-*Bacteroidetes* ratio relative to GM1. Meanwhile, GM1 was enriched for *Patescibacteria*, *Tenericutes,* and *Verrucomicrobia* (Fig. 3c). Overall, 60 OTUs were determined to be differentially abundant between GM1 and GM4 based on a False Discovery Rate (FDR) < 0.05 (Table S1). A heat map was used to show fold-change of the 25 most significantly modulated OTUs, and further describes the distinct nature of GM1 and GM4 through hierarchical clustering analysis (Fig. 3d). To summarize, GM1 and GM4 are distinct complex GM communities at the phylum and OTU level, and confer differential susceptibility to adenoma development in B6NB6JMF1-*Apc*^*Min*^ mice.

## Discussion

For almost three decades, the Min mouse has served as one of the most widely used animal disease models, providing a wealth of information about underlying mechanisms and potential therapeutic targets in CRC. The quantitative nature of the Min phenotype has been particularly useful for identifying potential modifiers of adenoma initiation, and evaluating response to prevention and therapeutic interventions. In this study, we aimed to determine whether two related inbred genetic strains, C57BL/6J and C57BL/6N, differentially modulate the Min adenoma phenotype, and to establish whether distinct GM communities could modify the Min phenotype. In doing so, we provide a platform for the discovery of novel genetic and microbial modifiers of Min, and demonstrate the multifaceted determinants of adenoma initiation comprising the commonly observed phenotypic variability associated with the Min mouse.

We crossed C57BL/6JM-*Apc*^*Min*^ (B6/JM) males from the original Min colony from the McArdle Laboratory at the University of Wisconsin with C57BL/6J (B6/J) females to generate a B6JB6JMF1 Min cohort (Fig. 1). We also used C57BL/6N (B6/N) females to generate a B6NB6JMF1 Min cohort, such that we could compare the two F1 groups to determine their modulatory effects on adenoma initiation. Furthermore, B6NB6JMF1 embryos were rederived using surrogate dams harboring two different GMs (Fig. 2a): GM1 resembles the GM of B6JB6JMF1 mice, while GM4 represents a distinct community (Fig. 3a), thereby enabling interrogation of the GM as an environmental modifier of Min (Hart et al. 2018). We found that the B6/J background conferred moderate suppression of the original B6/JM adenoma phenotype, resulting in an intermediate F1 phenotype relative to parental strains (Fig. 1) (colonic adenoma counts, Fig. S1). We also observed that both GM1- and GM4-colonized B6NB6JMF1 mice developed fewer adenomas than B6JB6JMF1 animals (Fig. 2b), suggesting that the B6/N background has a significantly stronger capacity to suppress adenomas than the B6/J background. Despite the relative relatedness of the B6/J and B6/N strains, these results suggest that during the course of their divergence, each strain has acquired variants that confer differential susceptibility to adenoma initiation. A number of studies have described phenotypic differences between B6/J and B6/N related to behavior, metabolism, and immunity. These changes are largely attributed to the approximated 150 homozygous SNPs detected between the substrains, including the classically referenced mutation in the nicotinamide nucleotide transhydrogenase (*Nnt*) gene in B6/J (Bryant 2011; Fontaine and Davis 2016). Given that these substrains have accumulated variants that differentially modify the Min phenotype, documented genomic differences between B6/J and B6/N can be used as a platform to identify additional modifiers of Min.

In addition to genetic divergence between the B6/J and B6/N substrains, we cannot rule out the possibility of maternal influences with respect to adenoma multiplicity. In this experimental design, B6JB6JMF1 mice were born through natural mating while B6NB6JMF1_GM1_ and B6NB6JMF1_GM4_ were rederived using surrogate dams. Thus, it is possible that in utero conditions or post-partum maternal care influenced SI and colon adenoma counts differentially in B6JB6JMF1 and B6NB6JMF1 mice. Future studies in which B6JB6JMF1 are also rederived using GM1 and GM4 surrogates are therefore necessary to resolve the effects of maternal care and genetic substrain differences. Regardless, these results provide evidence that investigators should be highly cognizant of specific substrains present in their colonies, and of changes in maternal care through common procedures such as cross fostering and rederivation when considering complex trait analyses.

While both GM1- and GM4-colonized B6NB6JMF1 mice had fewer adenomas than B6JB6JMF1 mice, we noted a significant modulatory effect of the GM, where GM4-colonized mice developed more adenomas than their GM1 counterparts (Fig. 2b) (Colonic adenoma counts, Fig. S2). Thus, Min mice with genetically identical backgrounds may have phenotypic variability based on GM colonization. Next-generation sequencing (NGS) of the microbial 16S rRNA gene enables identification and relative quantification of all detected operational taxonomic units (OTUs) (Ward et al. 1990). Given the apparent phenotypic effects of the GM, we utilized this approach to characterize the GMs of all F1 mice, as well as the B6/J and B6/JM parental colonies. We subsequently performed Principal Component Analysis (PCA) to visualize β-diversity and thus infer similarity of overall GM community structure between different groups (Fig. 3a). As mentioned previously, PCA demonstrates the relative similarity of the GMs of B6JB6JMF1 and B6NB6JMF1_GM1_ mice. Thus, we adequately controlled for GM variability when comparing the two groups. PCA also demonstrates the distinct nature of GM1 and GM4 that resulted in differential adenoma susceptibility, as anticipated based on the surrogate dams used for rederivation. Further analysis of GM1 and GM4 revealed differentially abundant phyla, which included a significant increase in the *Firmicutes:Bacteroidetes* ratio in GM4 relative to the GM1 (Fig. 3b). Interestingly, previous reports analyzing samples from human IBD and CRC patients have found similar trends when compared to healthy controls (Bamola et al. 2017). We also noted enrichment of several members of the *Proteobacteria* phylum, including sulfidogenic *Desulfovibrio* and *Bilophila* sp (Fig. 3d). Though various studies have suggested a potentially carcinogenic role for these bacteria (Attene-Ramos et al. 2006; Guo et al. 2016; Hellmich and Szabo 2015), studies that directly interrogate their phenotypic influence in the context of a complex GM are required. Of note, we also found that the GM of B6JB6JMF1 mice was distinct from many samples collected from the B6/J and B6/JM parental colonies. This may be explained by the fact that the sequenced B6/J and B6/JM fecal samples were not from the immediate parents of the F1 cohort, but were rather extracted from various generations of the parental colonies. Notably, a small number of parental colony fecal samples did cluster with the F1 cohort. This suggests that GM drift likely occurred over the course of several generations of maintaining the parental B6/J and B6/JM parental colonies. Investigators should therefore remain mindful of these potential changes, particularly when analyzing quantitative phenotypes that may be subject to the GM.

The Min mouse exhibits a classic quantitative trait phenotype with various biological systems contributing to composite adenoma multiplicity. This model has particularly served those in the genetics community seeking to identify genetic modifiers of adenoma susceptibility. In this study, we demonstrate differential adenoma suppression mediated by the B6/J and B6/N genetic backgrounds, establishing the need for investigators to be highly attentive to specific substrains when interpreting quantitative trait analyses. Furthermore, we show that the GM may also modify complex traits such as Min adenoma multiplicity. Thus, we not only provide a platform for the discovery of novel genetic and microbial modifiers of Min, but also establish a complex combination of physiological determinants comprising quantitative trait outcomes.

## Materials and methods

### Ethics statement

Animal studies were conducted in an Association for Assessment and Accreditation of Laboratory Animal Care International (AAALAC) accredited facility based on the guidelines provided by the Guide for the Care and Use of Laboratory Animals. All animal studies were approved by the University of Missouri Institutional Animal Care and Use Committee.

### Animals

Frozen C57BL/6JMlcr-*Apc*^*Min*^/Mmmh (B6/JM) embryos from the original Min colony at McArdle Laboratory, University of Wisconsin were acquired and rederived in our facility at the University of Missouri using CD-1 surrogate dams with a GM representing The Jackson Laboratory (Crl:CD1_GM1_), previously generated in our laboratory (Hart et al. 2018) (Taxon ID 10090). C57BL/6J-*Apc*^*Min*^ (B6/J) was acquired from the Jackson Laboratory. Both B6/JM and B6/J colonies were maintained at the University of Missouri as separate colonies. To generate B6JB6JMF1-*Apc*^*Min*^ mice, 6–8-week-old B6/J-*Apc*^+*/*+^ females were mated with 6–8-week-old B6/JM-*Apc*^+/*Min*^ males. Four- to five-week-old female C57BL/6NHsd (B6/N) were purchased from Envigo (Indianapolis, IN). To generate B6NB6JMF1-*Apc*^+*/Min*^ mice, in vitro fertilization (IVF) was performed as described previously using 5- to 8-week-old B6/N females and B6/JM males (Takeo 2011). Presumed zygotes were then placed in a KSOM dish followed by incubation for 24 h to allow advancement to the two-cell stage (Biggers and Raffin 2000). In order to establish B6NB6JMF1-*Apc*^+*/Min*^ with two distinct complex GMs, two cohorts of CD-1 surrogate dams were used as embryo transfer recipients to perform complex microbiota targeted rederivation (CMTR) (Hart et al. 2018). CD-1 females harboring a GM (Hsd:CD1_GM4_) were previously acclimated and maintained as a colony at the University of Missouri prior to their use as surrogates. The aforementioned Crl:CD1_GM1_ females harboring a GM representing The Jackson Laboratory were used as the second cohort, such that half of the B6NB6JMF1-*Apc*^*Min*^ embryos were transferred into CD-1_GM1_ surrogates, and half into CD-1_GM4_ surrogates. All female CD-1 embryo recipients were mated with vasectomized CD-1_GM1_ or CD-1_GM4_ males, respectively, and those that were copulatory plug-positive were used for embryo transfer. CD-1 females were anesthetized via IM injection of ketamine/xylazine cocktail at 5.5 mg and 1 mg per 100 g body weight, respectively. A dorsal midline incision was made and the uterine oviducts located by dissecting through the retroperitoneal muscle. Embryos in 3 to 5 µL of media were injected into the oviducts using a glass hand-pipette. Skin incisions were closed with sterile surgical staples and mice received a subcutaneous injection of 2.5 mg/kg of body weight flunixin meglumine (Banamine^®^) prior to recovery on a warming pad.

Surrogate dams were allowed to naturally deliver pups such that resulting B6NB6JMF1-*Apc*^*Min*^ would acquire the GM of their respective surrogate dams. Thus, these mice are denoted as ${\text{B6NB6JMF1-}}{Apc^{Min}}_{\text{GM1}}$ and ${\text{B6NB6JMF1-}}{Apc^{Min}}_{\text{GM4}}$. All mice were group-housed according sex, genetic lineage, and acquired GM (CMTR pups) in micro-isolator cages on ventilated racks (Thoren, Hazelton, PA). All mice had ad libitum access to 5058 irradiated breeder chow (LabDiet, St. Louis, MO) and acidified autoclaved water, maintained on paper chip bedding (Shepherd Specialty Papers, Watertown, TN) with 14:10 light:dark cycle. For genotyping, B6/J, B6/JM, and all F1 mice were ear-punched at 21 days of age (weaning). DNA was extracted using the “HotSHOT” genomic DNA preparation method as described (Truett et al. 2000).

### Adenoma counts

At 100 days of age, mice requiring adenoma counts were euthanized via CO_2_ asphyxiation. Following exposure through the abdominal cavity, whole small and large intestines were incised longitudinally, flushed with saline and placed on bibulous paper with the luminal side facing up for formalin fixation. Grossly visible adenomas were counted manually using a Leica M165FC microscope at ×12.5 magnification.

### Sample collection and DNA extraction for 16S rRNA sequencing

Fecal samples were collected from B6/J, B6/JM, B6JB6JMF1, and both CMTR-derived B6NB6JMF1 groups at 1 month of age. Mice were placed in an empty autoclaved cage until they defecated naturally. Two fecal pellets per mouse were collected aseptically and placed in a 2-mL round-bottomed tube containing 800 µL of lysis buffer as described (Ericsson et al. 2015) and a 0.5-cm-diameter stainless steel bead (Grainger, Lake Forest, Il). Fecal samples were mechanically disrupted using a TissueLyser II (Qiagen, Venlo, Netherlands) for 3 min at 45 Hz, followed by incubation at 70 °C for 20 min with periodic vortexing. DNA extraction from fecal pellets, cecal contents, and ileal epithelium for 16S rRNA sequencing was performed using a DNeasy Blood & Tissue Kit^®^ (Qiagen) as previously described (Ericsson et al. 2015).

## 16S library preparation and sequencing

DNA extracted from fecal pellets was sent to the University of Missouri DNA Core facility (Columbia, MO) for further processing. An amplicon library of the hypervariable V4 region of the bacterial 16S rRNA gene was generated using the U515F/806R primer set as described previously (Caporaso et al. 2010). Amplicons were sequenced on the Illumina MiSeq platform under the described conditions (Montonye et al. 2018).

### Informatics processing

Trimming, assembly, binning, and annotation of DNA sequences were performed at the University of Missouri Informatics Research Core Facility (Columbia, MO). Quality control of DNA, assembly of contiguous sequences, sequence removal after trimming for base quality, and chimera removal were completed as described (Montonye et al. 2018). All remaining sequences were assigned to operational taxonomic units (OTUs) by de novo OTU clustering based on 97% nucleotide similarity. OTUs were annotated using BLAST (Altschul et al. 1997) against the SILVA database (Ritari et al. 2015) of 16 s rRNA sequences and taxonomy. OTU relative abundances were subject to a ¼ root transformation prior to visualization using Principal Component Analysis (PCA). PCA visualization and α-diversity indices (Chao1 and Shannon) were acquired using Past 3.12 software (Hammer et al. 2001). Open access Metaboanalyst 3.0 was used to generate heat maps of microbiome data based on Euclidian distance measurements and the Ward clustering algorithm applied to the cube root-transformed dataset.

### Statistical analysis

Statistical analyses of all adenoma counts were performed using Analysis of Variance (ANOVA) with the Student Newman–Keul post hoc test in Sigma Plot 14.0 (Systat Software Inc., Carlsbad CA). For statistical comparisons of bacterial phyla and α-diversity indices, the student’s *t* test was used. Metaboanalyst 3.0 was used to determine statistically significant OTUs via the Student’s *t* test. For adenoma counts, phyla, and α-diversity, *p* values < 0.05 were considered statistically significant, while FDR values < 0.05 were considered significantly different for bacterial OTUs. GraphPad Prism 8 was used to generate all scatter plots, bar graphs, and box plots.

## Electronic supplementary material

Below is the link to the electronic supplementary material.

Supplementary material 1 (DOCX 79 kb)
