# Supplementary material for: The gut microbiota modulates differential adenoma suppression by B6/J and B6/N genetic backgrounds in ApcMin mice
Source: Mamm Genome. 2019 Sep 23;30(9):237–44. doi: 10.1007/s00335-019-09814-3 (PMC6842652; doi:10.1007/s00335-019-09814-3)
Supplement: Supplementary file 1 — Supplementary material 1 (DOCX 79 kb) [file 335_2019_9814_MOESM1_ESM.docx]

**SUPPLEMENTARY FIGURE LEGENDS**

**Figure S1. Colonic adenoma numbers in B6/J and B6/JM parental lines and B6JB6JMF1-*Apc^Min^* offspring.** Scatter plots comparing mean (± SD) colonic adenoma counts of the original B6-*Apc^Min^* colony generated at UW McArdle Laboratory (B6/JM), B6-*Apc^Min^* mice acquired from The Jackson Laboratory and maintained at University of Missouri (B6/J), and their F1 offspring (B6JB6JMF1) (B6/JM, n = 19; B6/J, n = 22; B6JB6JMF1, n = 19). ANOVA with the Student Newman-Keuls method.

**Figure S2. Colonic adenoma counts in rederived F1 *Apc^Min^* mice.** Scatter plots comparing mean (± SD) colonic adenoma counts of B6JB6JMF1-*Apc^Min^*, B6NB6JMF1-*Apc^Min^*_GM1_, and B6NB6JMF1-*Apc^Min^*_GM4_ mice. ANOVA with the Student Newman-Keuls method.

**SUPPLEMENTARY FIGURES**


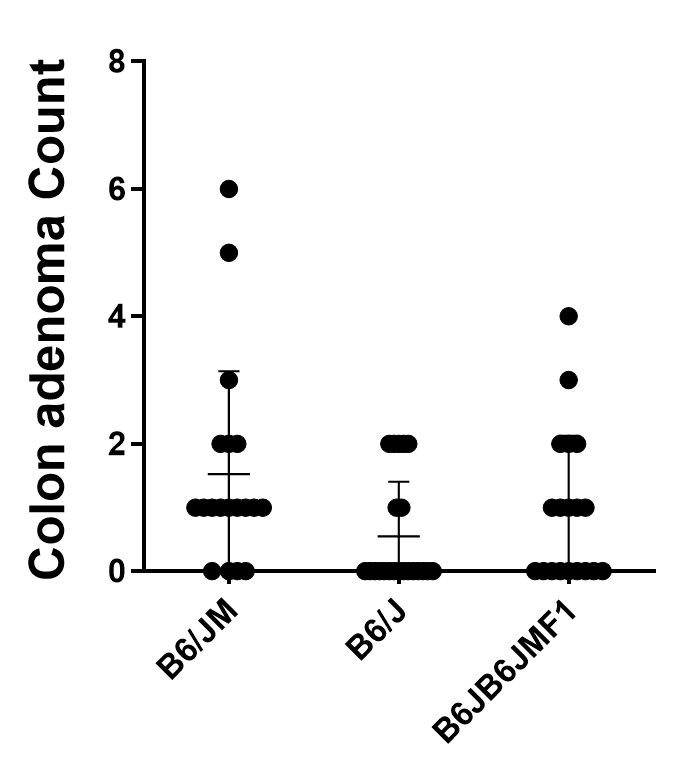
**Figure S1**

**Figure S2**


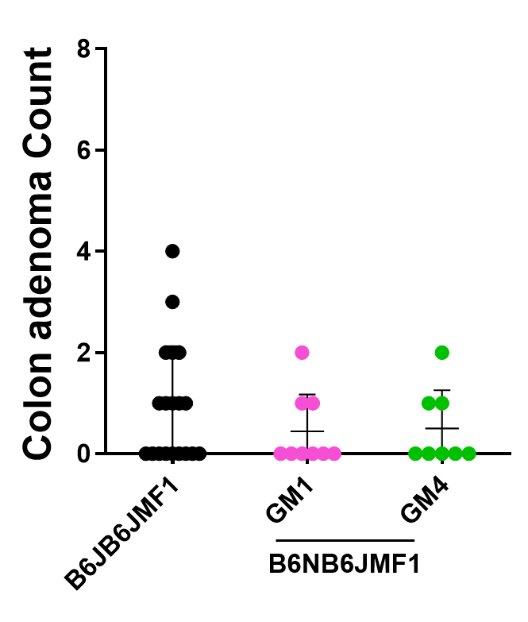


**SUPPLEMENTARY TABLES**

**Table S1. Significantly different fecal OTUs between GM1 and GM4**

| **Significant OTUs in B6NB6JMF1 mice (FDR < 0.05)** | | **GM1**  **relative to GM4** | | **GM1** | **GM4** |
| --- | --- | --- | --- | --- | --- |
| **Phylum** | **OTU** | **FDR** | **Mean Fold Change** | **Mean Relative Abundance (±SEM)** | **Mean Relative Abundance (±SEM)** |
| *Bacteroidetes* | genus *Odoribacter*.1 | NA | NA | 0.00554±0.0006 | ND |
| *Bacteroidetes* | genus *Odoribacter* | NA | NA | ND | 0.0027±0.0004 |
| *Bacteroidetes* | genus *Alloprevotella*.1 | NA | NA | ND | 0.0326±0.0048 |
| *Bacteroidetes* | genus *Rikenella* | NA | NA | ND | 0.0026±0.0004 |
| *Bacteroidetes* | genus *Rikenellaceae* RC9 gut group | NA | NA | ND | 0.0020±0.0003 |
| *Tenericutes* | order *Mollicutes* RF39.1 | 3.69E-12 | 1342.3 | 0.0071±0.0012 | 2.66E-06±1.47E-06 |
| *Verrucomicrobia* | genus *Akkermansia* | NA | NA | 0.0003  ±5.93E-05 | ND |
| *Firmicutes* | genus *Lachnospiraceae* NK4A136 group.3 | 3.41E-11 | 0.002448 | 0.0399±0.0074 | 0.1325±0.0234 |
| *Bacteroidetes* | genus *Rikenellaceae* RC9 gut group.1 | 5.21E-10 | 0.012343 | 3.77E-06±2.03E-06 | 0.0005±9.30E-05 |
| *Proteobacteria* | genus *Bilophila* | NA | NA | ND | 0.0012±0.0002 |
| *Firmicutes* | genus *Lachnospiraceae* UCG-009 | 4.41E-09 | 35.276 | 0.0003±6.079E-05 | 5.79E-06±3.95E-06 |
| *Proteobacteria* | genus *Desulfovibrio*.1 | NA | NA | ND | 0.0058±0.0012 |
| *Firmicutes* | genus *Erysipelatoclostridium* | NA | NA | ND | 7.82E-05±1.33E-05 |
| *Bacteroidetes* | genus *Bacteroides*.1 | 9.30E-09 | 7.2568 | 0.1031±0.0102 | 0.0138±0.0030 |
| *Firmicutes* | genus *Butyricicoccus*.1 | NA | NA | 0.0021±0.0005 | ND |
| *Deferribacteres* | genus *Mucispirillum* | NA | NA | ND | 0.0082±0.0024 |
| *Firmicutes* | genus *Tyzzerella* 4 | NA | NA | ND | 0.0003±7.12E-05 |
| *Firmicutes* | order *Clostridiales*.1 | 2.23E-07 | 10.095 | 0.0002±2.52E-05 | 1.59E-05±5.16E-06 |
| *Proteobacteria* | genus *Desulfovibrio* | NA | NA | ND | 0.0010±0.0003 |
| *Firmicutes* | genus *Roseburia*.1 | 5.09E-06 | 60.418 | 0.0022±0.0008 | 3.89E-05±2.14E-05 |
| *Firmicutes* | genus *Intestinimonas* | 6.99E-06 | 0.043832 | 3.17E-06±1.51E-06 | 0.0001±2.90E-05 |
| *Firmicutes* | genus *Eubacterium nodatum* | 7.53E-06 | 3.7466 | 0.0005±4.57E-05 | 0.0001±3.04E-05 |
| *Firmicutes* | genus *Eubacterium oxidoreducens* | 1.40E-05 | 469.53 | 0.0018±0.0006 | 1.78E-06±1.32E-06 |
| *Cyanobacteria* | order *Gastranaerophilales*.1 | NA | NA | ND | 0.0005±0.0002 |
| *Firmicutes* | genus *Ruminiclostridium* 6.1 | NA | NA | ND | 0.0015±0.0005 |
| *Firmicutes* | family *Lachnospiraceae*.5 | 2.69E-05 | 91.759 | 0.0032±0.0011 | 2.92E-05±8.47E-06 |
| *Firmicutes* | genus *Lachnospiraceae* NC2004 group | NA | NA | ND | 0.0004±0.0001 |
| *Bacteroidetes* | family *Muribaculaceae*.1 | 3.82E-05 | 5.0969 | 0.0001±1.52E-05 | 1.73E-05±4.44E-06 |
| *Proteobacteria* | genus *Parasutterella* | NA | NA | ND | 5.76E-05±1.45E-05 |
| *Bacteroidetes* | family *Muribaculaceae* | 8.97E-05 | 0.45146 | 0.0021±0.0002 | 0.0047±0.0005 |
| *Firmicutes* | order *Clostridiales*.2 | NA | NA | ND | 8.11E-05±2.39E-05 |
| *Patescibacteria* | genus *Candidatus Saccharimonas*.1 | 0.000149 | 5.9624 | 0.0048±0.0010 | 0.0008±0.0002 |
| *Patescibacteria* | genus *Candidatus Saccharimonas* | 0.000333 | 3.8575 | 0.0083±0.0014 | 0.0021±0.0004 |
| *Actinobacteria* | genus *Bifidobacterium* | 0.000365 | 0.009988 | 1.70E-06±1.23E-06 | 0.0004±0.0001 |
| *Firmicutes* | family *Erysipelotrichaceae*.1 | 0.000381 | 9.0356 | 0.0002±5.18E-05 | 2.79E-05±8.53E-06 |
| *Bacteroidetes* | genus *Parabacteroides* | 0.000696 | 2.5102 | 0.0010±0.0001 | 0.0003±7.70E-05 |
| *Firmicutes* | genus *Ruminococcaceae* UCG-014 | 0.000775 | 3.8747 | 0.0013±0.0003 | 0.0003±0.0002 |
| *Firmicutes* | genus *Ruminococcaceae* UCG-010.2 | 0.000775 | 2.1063 | 0.0004±4.25E-05 | 0.0002±2.73E-05 |
| *Bacteroidetes* | genus *Prevotella* 9 | 0.000803 | 3.2998 | 0.0086±0.0011 | 0.0025±0.0007 |
| *Firmicutes* | *Lactobacillus gasseri* | 0.000849 | 0.14097 | 9.59E-05±3.80E-05 | 0.0007±0.0002 |
| *Tenericutes* | genus *Anaeroplasma* | NA | NA | 0.0017±0.0006 | ND |
| *Firmicutes* | genus *Blautia*.2 | NA | NA | ND | 6.10E-05±2.38E-05 |
| *Firmicutes* | genus *Ruminiclostridium* | 0.00122 | 0.012429 | 4.25E-06±1.99E-06 | 0.0005±0.0002 |
| *Firmicutes* | family *Christensenellaceae*.1 | 0.002353 | 2.6456 | 0.0002±2.48E-05 | 7.57E-05±1.75E-05 |
| *Firmicutes* | genus *Tyzzerella* 3 | NA | NA | 0.0001±6.01E-05 | ND |
| *Firmicutes* | genus *Lachnospiraceae* NK4A136 group.2 | 0.002871 | 0.30295 | 1.75E-05±7.60E-06 | 0.0087±0.0012 |
| *Firmicutes* | genus *Oscillibacter*.1 | 0.002998 | 0.24738 | 0.0019±0.0004 | 0.0080±0.0017 |
| *Proteobacteria* | genus *Oxalobacter* | 0.004546 | 2.794 | 0.0002±2.87E-05 | 4.84E-05±1.58E-05 |
| *Firmicutes* | genus *Lachnospiraceae* UCG-010 | 0.005379 | 8.7386 | 0.0001±3.30E-05 | 1.13E-05±4.30E-06 |
| *Firmicutes* | genus *Lachnoclostridium*.1 | 0.005824 | 5.1709 | 0.0033±0.0009 | 0.0007±0.0002 |
| *Firmicutes* | family *Clostridiales* vadinBB60 group.2 | 0.006673 | 0.44348 | 0.0014±0.0003 | 0.0033±0.0005 |
| *Firmicutes* | genus *Intestinimonas*.1 | 0.011292 | 0.21214 | 5.41E-05±1.22E-05 | 0.0002±6.11E-05 |
| *Firmicutes* | genus *Harryflintia*.1 | 0.011569 | 0.27058 | 2.64E-05±8.10E-06 | 9.83E-05±2.50E-05 |
| *Firmicutes* | family *Lachnospiraceae*.4 | 0.012257 | 3.6923 | 0.0015±0.0004 | 0.0004±7.30E-05 |
| *Firmicutes* | genus *Ruminococcus* 1 | 0.013196 | 0.22256 | 6.74E-05±1.39E-05 | 0.0003±8.96E-05 |
| *Firmicutes* | genus *Tyzzerella* | NA | NA | ND | 8.56E-06±2.78E-06 |
| *Firmicutes* | family *Ruminococcaceae* | 0.021464 | 0.3296 | 2.09E-05±5.34E-06 | 5.70E-05±1.11E-05 |
| *Firmicutes* | genus *Marvinbryantia* | 0.022038 | 0.22811 | 6.54E-05±1.38E-05 | 0.0003±7.89E-05 |
| *Firmicutes* | genus *Acetatifactor* | 0.023512 | 7.8109 | 0.0005±0.0002 | 6.58E-05±1.78E-05 |
| *Firmicutes* | family *Erysipelotrichaceae* | 0.044132 | 2.691 | 0.0007±0.0002 | 0.0003±4.32E-05 |

ND: Relative abundance was below detectable limits by 16S sequencing

NA: Statistical comparison was not applicable due to a relative abundance below detectable limits (ND) in at least one GM group
